# Gray matter covariations in autism: out-of-sample replication using the ENIGMA autism cohort

**DOI:** 10.1186/s13229-024-00583-8

**Published:** 2024-01-17

**Authors:** Ting Mei, Alberto Llera, Natalie J. Forde, Daan van Rooij, Dorothea L. Floris, Christian F. Beckmann, Jan K. Buitelaar

**Affiliations:** 1 Department of Cognitive Neuroscience, Donders Institute for Brain, Cognition and Behaviour, Radboud University Medical Centre, Kapittelweg 29, 6525EN Nijmegen, The Netherlands; 2 Department of Psychology, Utrecht University, Utrecht, The Netherlands; 3 Methods of Plasticity Research, Department of Psychology, University of Zurich, Zurich, Switzerland; 4 Centre for Functional MRI of the Brain, University of Oxford, Oxford, UK; 5 Karakter Child and Adolescent Psychiatry University Centre, Nijmegen, The Netherlands

**Keywords:** Autism, Gray matter volume covariation, Replication

## Abstract

**Background:**

Autism spectrum disorder (henceforth autism) is a complex neurodevelopmental condition associated with differences in gray matter (GM) volume covariations, as reported in our previous study of the Longitudinal European Autism Project (LEAP) data. To make progress on the identification of potential neural markers and to validate the robustness of our previous findings, we aimed to replicate our results using data from the Enhancing Neuroimaging Genetics Through Meta-Analysis (ENIGMA) autism working group.

**Methods:**

We studied 781 autistic and 927 non-autistic individuals (6–30 years, IQ ≥ 50), across 37 sites. Voxel-based morphometry was used to quantify GM volume as before. Subsequently, we used spatial maps of the two autism-related independent components (ICs) previously identified in the LEAP sample as templates for regression analyses to separately estimate the ENIGMA-participant loadings to each of these two ICs. Between-group differences in participants’ loadings on each component were examined, and we additionally investigated the relation between participant loadings and autistic behaviors within the autism group.

**Results:**

The two components of interest, previously identified in the LEAP dataset, showed significant between-group differences upon regressions into the ENIGMA cohort. The associated brain patterns were consistent with those found in the initial identification study. The first IC was primarily associated with increased volumes of bilateral insula, inferior frontal gyrus, orbitofrontal cortex, and caudate in the autism group relative to the control group (*β* = 0.129, *p* = 0.013). The second IC was related to increased volumes of the bilateral amygdala, hippocampus, and parahippocampal gyrus in the autism group relative to non-autistic individuals (*β* = 0.116, *p* = 0.024). However, when accounting for the site-by-group interaction effect, no significant main effect of the group can be identified (*p* > 0.590). We did not find significant univariate association between the brain measures and behavior in autism (*p* > 0.085).

**Limitations:**

The distributions of age, IQ, and sex between LEAP and ENIGMA are statistically different from each other. Owing to limited access to the behavioral data of the autism group, we were unable to further our understanding of the neural basis of behavioral dimensions of the sample.

**Conclusions:**

The current study is unable to fully replicate the autism-related brain patterns from LEAP in the ENIGMA cohort. The diverse group effects across ENIGMA sites demonstrate the challenges of generalizing the average findings of the GM covariation patterns to a large-scale cohort integrated retrospectively from multiple studies. Further analyses need to be conducted to gain additional insights into the generalizability of these two GM covariation patterns.

**Supplementary Information:**

The online version contains supplementary material available at 10.1186/s13229-024-00583-8.

## Background

Autism spectrum disorder (autism) is a neurodevelopmental condition characterized by social-communicative difficulties, accompanied by restrictive and repetitive behaviors, and altered sensory processing [2]. The lifelong impact of autism prompts research into the etiology underpinning this condition. In the exploration of the brain substrates of autism, many anatomical magnetic resonance imaging (MRI) studies have reported that autism is associated with differences in gray matter (GM) morphology (e.g., [28]). However, divergent findings of GM morphometry have been demonstrated in multiple brain regions (e.g., [6, 11]) complicating the identification of neural correlates of autism. For example, former large-scale studies found either smaller or no changed volume with respect to the subcortical areas (e.g., pallidum) in autism [28, 31]. Furthermore, previous research reported increased or decreased volume in several cortical areas (e.g., right inferior temporal gyrus) [6, 11].

We inferred previously that divergent findings can partly be attributed to the appliance of mass univariate approaches in these studies while not considering the structural GM covariations in the brain [22]. Our previous work introduced independent component analysis (ICA) on voxel-based morphometry (VBM) data in a deeply phenotyped large autism sample, the EU-AIMS Longitudinal European Autism Project (LEAP) [8, 20]. There we demonstrated that there are two autism-related brain GM covariation patterns involving (1) insula, frontal areas, and caudate, and (2) amygdala, hippocampus, and parahippocampal gyrus (PHG). These findings corroborated our inferences that autism is associated with the combined influence of several GM regions, and our subsequent study additionally supported the stability of the GM covariation patterns [21]. The ICA approach sensitized our analysis to GM covariation patterns in the data rather than regional differences like more traditional methods. This allowed us to identify small between-group effects contributing to progress in the identification of neural markers of autism. However, in addition to the data analytic strategy, the high neurobiological and phenotypic heterogeneity of autism produces obstacles to the generalization of a common GM covariation pattern of autism. Accordingly, validation across cohorts is crucial to make headway in identifying robust diagnostic markers that can facilitate the understanding of autism etiology.

Consequently, to test the robustness and validity of our previous findings, in this study we aimed to replicate our previous results using a large independent dataset—the Enhancing Neuroimaging Genetics Through Meta-Analysis (ENIGMA) autism cohorts. This collaboration was established to unify preprocessing and analytic approaches worldwide, and aggregate the genetic and neuroimaging data of autistic and control individuals of all ages [28]. Within the framework of the present study, we adhered closely to the analytical pipeline of the original study. We utilized the spatial maps of autism-related GM covariation patterns identified in the LEAP dataset [22] to extract the relevant participant loadings from individuals within the ENIGMA dataset. Thereafter we explored the case–control difference in GM covariations in ENIGMA. We hypothesized that the GM covariation patterns applied to an independent dataset would also show case–control differences—thus showing the generalizability of autism-related spatial maps of LEAP to other autism cohorts.

## Methods and materials

### Participants

The participants were part of the ENIGMA autism cohort (http://enigma.ini.usc.edu/ongoing/enigma-asd-working-group), which aims at assembling MRI data across a wide range of autism studies. We refer to [28] for a detailed description of this cohort. The working group implemented a data freeze in December 2020, at which point there were 1833 autistic individuals and 1838 non-autistic individuals (aged 2–64 years old) from 56 sites. All individuals with autism were included based on clinical diagnosis according to DSM-IV or DSM-5. In correspondence with the inclusion and exclusion criteria of our previous study on the LEAP sample [22], we included participants with available T1-weighted images, aged 6–30 years old, with available IQ data and an IQ ≥ 50 (*n* = 1978). All T1 images were checked visually and participants were excluded due to structural abnormalities (e.g., enlarged ventricles, part of brain issues missing, and distortions), or excessive head motion (*n* = 31). This resulted in 905 autistic and 1042 non-autistic individuals from 37 sites entering the preprocessing pipeline. Local ethical approval was acquired in each participating site.

### VBM estimation

The acquisition parameters of T1-weighted images at each site can be found in Additional file 1: Table S1. We implemented the same preprocessing pipeline as previously [22] to estimate the VBM images of each participant using the CAT12 toolbox (https://dbm.neuro.uni-jena.de/cat/, accessed 25 Jan 2022) in SPM12 (Wellcome Department of Imaging Neuroscience, London, UK). All T1-weighted images were first segmented into GM, white matter, and cerebrospinal fluid, which were then affinely registered to the MNI template. We normalized all segmented GM maps to MNI space using the previously customized group DARTEL (high-dimensional, nonlinear diffeomorphic registration algorithm, [1]) template in LEAP generated on LEAP sample [22]. Finally, all images were smoothed with a 4 mm full-width half-max (FWHM) isotropic Gaussian kernel. A full quality control check was applied containing calculations of quantitative quality measures based on the CAT12 pipeline that includes mean correlation from the sample homogeneity module and weighted overall image quality ratings (IQR), and visualizations of the segmentations.

### Construction of independent sources of spatial variation

**Fig. 1:**
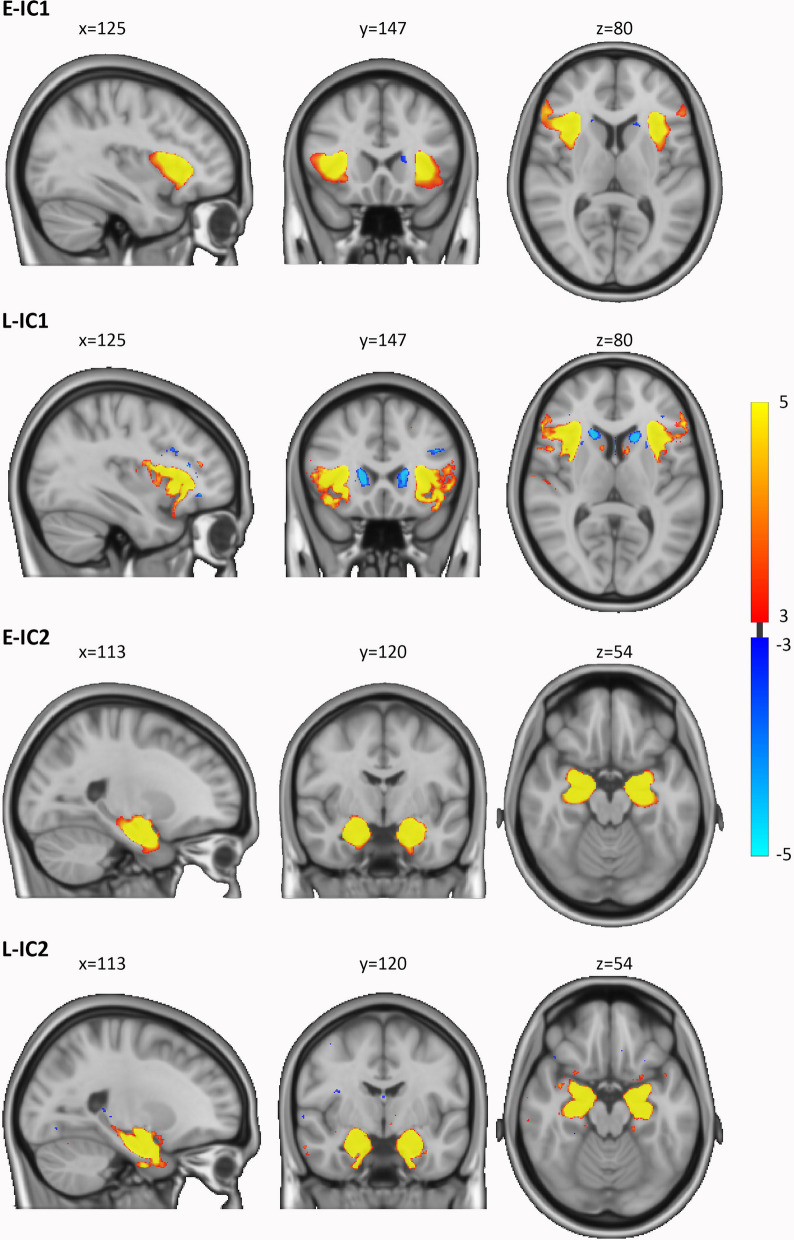
The spatial maps of the two components of interest (i.e., group differences) in the current and initial study. The spatial maps were thresholded at 3 <|*z*|< 5. *E-IC* independent component of ENIGMA, *L-IC* independent component of LEAP

In our previous study, we utilized ICA to decompose all participants’ VBM images into 100 spatially independent sources (or components, ICs) of spatial variation, two of which were found to be significantly related to autism (we refer these two ICs as L-IC1, L-IC2, Fig. 1). Aligning with the objectives of our current study, we performed linear regressions (Eqs. 2–4) to apply ICA-derived components to the ENIGMA VBM data. In essence, ICA operates on the premise of a linear regression model. Specifically, we generated the ENIGMA specific participant loadings based on knowing the decomposed ICs and the VBM maps of ENIGMA sample. To ensure the complete configuration estimated for the whole brain, we utilized the original set of 100 spatial IC patterns derived from LEAP in these linear regression analyses. The LEAP 100 ICs spatial maps were thresholded to the voxel values at |*z*|> 3. The participant loading in the current study refers to the individual contribution of each participant to each component. The VBM input images were voxel-wise demeaned and divided by the standard deviation across participants, and we subsequently implemented regression analyses as follows:

In Eq. (1), we represent the VBM ICA decomposition performed in the LEAP sample, which was implemented using MELODIC-ICA [4].

1 $$A_{{v \times s_{L} }}^{L} \sim B_{v \times i}^{L} \times C_{{i \times s_{L} }}^{L}$$

where $A^{L}$ is a matrix with all participants’ VBM images concatenated from LEAP, and $B^{L}$ and $C^{L}$ are matrices containing the estimated independent spatial maps and participants’ loadings to each IC, respectively. For completeness, *v* denotes the number of voxels on each VBM image, *s*_*L*_ represents the number of participants in LEAP, and *i* is the number of ICs (*i* = 100).

To obtain a primary representation of the participant loadings in ENIGMA with respect to the spatial maps from LEAP (matrix $B_{v \times i}^{L}$), we used the ENIGMA VBM data ($A^{E}$) and Moore–Penrose pseudoinverse [3] to calculate and extracted the corresponding spatial maps (two columns from $B_{v \times 100}^{E}$) and participant loadings (two rows from $\hat{C}_{{100 \times s_{E} }}^{E}$) of the two ICs of interest.

2 $$C_{{100 \times s_{E} }}^{E} = ((B_{v \times 100}^{L} )^{\prime} \times B_{v \times 100}^{L} )^{ - 1} \times (B_{v \times 100}^{L} )^{\prime} \times A_{{v \times s_{E} }}^{E} ;$$

3 $$B_{v \times 100}^{E} = A_{{v \times s_{E} }}^{E} \times (C_{{100 \times s_{E} }}^{E} )^{\prime} \times \left( {C_{{100 \times s_{E} }}^{E} \times \left( {C_{{100 \times s_{E} }}^{E} } \right){\prime} } \right);$$

4 $$\hat{C}_{{100 \times s_{E} }}^{E} = = ((B_{v \times 100}^{E} )^{\prime} \times B_{v \times 100}^{E} )^{ - 1} \times (B_{v \times 100}^{E} )^{\prime} \times A_{{v \times s_{E} }}^{E} ;$$

Equations 2–4 exhibit the three steps of regression analyses in ENIGMA that estimate the unknown matrices using the known ones based on matrix multiplications. That is, in Eq. (2) $C^{E}$ was estimated based on the transformation of matrix multiplication using known spatial maps of the 100 ICs from LEAP ($B^{L}$) and all participants’ VBM images from ENIMGA ($A^{E}$). Similarly, the estimated spatial maps of the two ICs of interest in ENIGMA ($B^{E}$) were calculated using $C^{E}$ and $A^{E}$. At last, the estimated $B^{E}$ was refitted to the linear model (Eq. 4) to compute the final participant loadings of the two ICs of interest in ENIGMA (two rows from $\hat{C}^{E}$) that were used in the further statistical analyses. The input spatial maps ($B^{L}$) were thresholded at |*z*|> 3.

### Statistical analyses

Dice similarity coefficients were used to quantify the overlap between the LEAP and generated ENIGMA spatial maps. For this, the maps were thresholded (|*z*|> 3) and binarized.

To validate our previous findings of autism-related independent sources of spatial variation, we used a generalized linear model (GLM) to investigate group differences of the two constructed ICs from ENIGMA while accounting for the effect of age, sex, IQ, and scanner site. Initial analysis revealed striking site effects which led us to implement ComBat [12, 14] before analysis in the GLM. The GLM after ComBat contained age, sex, IQ, and site as covariates as well. Subsequently, we performed a type-II analysis of deviance to check if site effects were still significant on the age-, sex- and IQ-corrected participant loadings after ComBat correction (see Additional file 1: Section 2). Moreover, to determine the consistency of findings across sites we incorporated a site-by-diagnosis interaction term as an additional covariate. We employed type-III analysis of deviance to evaluate the significance of the site-by-diagnosis interaction term on the two ICs of interest.

In the current dataset, the total score of the Autism Diagnostic Observation Schedule–Generic (ADOS) [18] was available in 486 autistic individuals (Additional file 1: Table S2), and it was used to examine the univariate relationship between the brain covariations and autistic trait. A GLM was employed to test the association while controlling for age, sex, IQ, and scanner site. Since there were limited additional clinical descriptive profiles available in the ENIGMA dataset (such as limited access to or availability of information on subscales of the Autism Diagnostic Interview-Revised [26] and the Autism Diagnostic Observational Schedule 2 [19]), we were unable to repeat the multivariate analysis between brain and behavior using canonical correlation analyses in the present study.

## Results

We excluded 5 participants due to segmentation failure. Furthermore, 218 participants were found to be potentially duplicate with each other as the correlations of their VBM images were unusually large (mostly *r* > 0.990), and therefore, these participants were excluded as well. Next, we visually checked the images with relatively extreme outliers of the image quality ratings (greater than three standard deviations from the sample mean). Accordingly, 32 participants required inspection, among which 18 participants were excluded due to insufficient data quality. Additionally, we excluded sites with less than 10 individuals to retain the statistical power within each site after preprocessing (3 sites were excluded, resulting in the exclusion of a total of 12 individuals). Consequently, there were 770 autistic individuals (667 males and 103 females, IQ > 50) and 924 non-autistic individuals (693 males and 231 females, IQ > 70) from 34 sites in the final sample (demographic information can be found in Table 1 and Additional file 1: Table S2).

**Table 1:** Characteristic of participants

Characteristic	Autism, *n* = 770			Controls, *n* = 924			*t*	*p* value
	Range	Mean	SD	Range	Mean	SD		
Age, years	6–30	14.88	5.47	6–30	14.79	5.75	0.33	0.742
IQ	57–149	106.22	16.91	71–149	113.42	12.73	− 9.73	*p* < 0.001
ADOS^a^	2–23	11.16	3.97	–				
		*n*	*%*		*n*	*%*	*χ*^***2***^	
Sex, male		667	86.62		693	75.00	35.85	*p* < 0.001

^a^There were 486 individuals with autism with available total ADOS score

### Group differences in the isolated two GM covariations

**Fig. 2:**
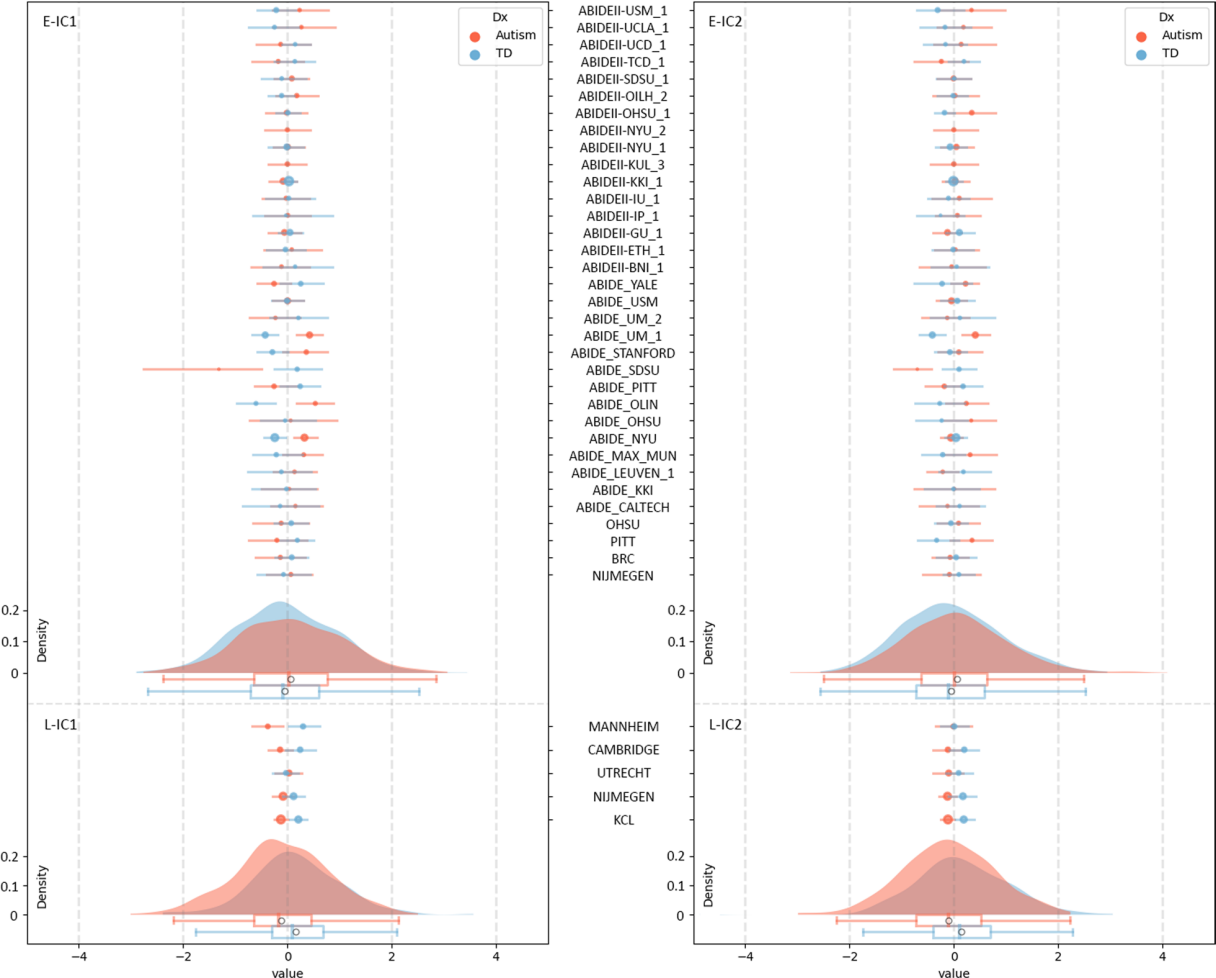
Distribution of the age-, sex-, IQ- and site-corrected (ComBat) participant loadings of the two components of interest across whole sample and within each site of ENIGMA. The forest plots demonstrate the mean and 95% confidential interval of each group in each site. The size of the mean marker ranges according to the sample size within each site for each group. The white circles in boxplots show the mean of each group across whole sample. *E-IC* independent component of ENIGMA, *L-IC* independent component of LEAP

The two components of interest (E-IC1, E-IC2), isolated in the ENIGMA dataset, showed significant case–control differences in participant loadings (E-IC1, *β* = 0.129, *p* = 0.013; E-IC2, *β* = 0.116, *p* = 0.024). The constructed brain patterns of these two components in the ENIGMA data are shown in Fig. 1, and these brain patterns showed high similarities with the L-IC maps both thresholded at |*z*|> 3 and binarized (dice similarity coefficient: IC1: 0.440, IC2: 0.519). The E-IC1 was primarily associated with varied volumes of bilateral insula, inferior frontal gyrus (IFG), orbitofrontal cortex (OFC), and mainly right caudate in the autism group relative to controls. The E-IC2 was related to altered volumes of the bilateral amygdala, hippocampus, and parahippocampal gyrus (PHG) in the autism group relative to controls. In the analysis incorporating the site-by-group effect, we excluded two sites that exclusively included autistic individuals (*n* = 42). The analysis revealed significant site-by-group effects for the two ICs of interest (E-IC1, *G*^2^ = 75.771, *p* < 0.001; E-IC2, *G*^2^ = 48.956, *p* = 0.021). When considering this interaction effect, the group effects no longer exhibited significance for either IC (E-IC1, *G*^2^ = 0.144, *p* = 0.704; E-IC2, *G*^2^ = 0.285, *p* = 0.593). Figure 2 shows that the group differences in each site were diverse, while the differences in the LEAP dataset remained consistent across sites. The group difference of the ICs in each site can be found in Additional file 1: Table S3. After accounting for the site-by-group effect, we were unable to identify a significant main effect of the group in the ENIGMA dataset. Consequently, the stability of the two autism-spatial patterns cannot be generalized to the ENIGMA sample. As for the application of ComBat to our data, we included diagnosis, age, sex, IQ as biological covariates in the analysis for sanity check. The results remain consistent with the original results. E-IC1 and E-IC2 were found with significant group effect (E-IC1, *β* = 0.127, *p* = 0.013; E-IC2, *β* = 0.119, *p* = 0.020). The group effects were not significant when considering the interaction effect of site and group (interaction effect, E-IC1, *G*^2^ = 74.938, *p* < 0.001; E-IC2, *G*^2^ = 50.839, *p* = 0.015; group effect, E-IC1, *G*^2^ = 0.133, *p* = 0.715; E-IC2, *G*^2^ = 0.233, *p* = 0.564).

At last, we did not find significant univariate associations between these two brain ICs and the total ADOS score in autism (E-IC1, *β* = − 0.911, *p* = 0.196; E-IC2, *β* = − 0.952, *p* = 0.086).

## Discussion

In the current study, we tested the robustness and validity of our previous findings [22] in another large independent sample of autistic and non-autistic individuals—the ENIGMA autism cohort. We estimated the case–control differences of brain covariation patterns in ENIGMA based on our previously identified autism-related GM covariation patterns in LEAP. In ENIGMA, we reproduced the GM covariation spatial configurations after thresholding included most of the regions in the insula, frontal areas, and caudate and the regions in the amygdala, hippocampus, and PHG. However, the autism-related effects of these two ICs were not significant after considering the effect of site-by-group interactions. The scanner sites in ENIGMA demonstrated diverse group differences. These two IC patterns therefore cannot be generalized in the current settings. Corresponding to the previous VBM studies, the volumes of these brain areas were found to either be decreased, increased, or unaltered in autistic individuals compared to non-autistic controls across different samples (e.g., [6, 11, 23]).

The overall non-significant results could be attributed to many factors. First, the large ENIGMA dataset is limited by large cross site differences including variation in scanner type, field strength and scan acquisition parameters. Second, site effects are not limited to scan acquisition parameters but also to variation in sample selection across sites. Different distribution of sample characteristics across sites, such as sex, cognitive level, or severity of autistic behaviors, was suggested as another potential confounder [29]. In LEAP, while still faced with a multi-site cohort, scan parameters were prospectively harmonized, sample demographics were matched across sites and identical testing protocols were utilized thereby reducing the confounding issues present in ENIGMA. Nevertheless, dual regression operates on an individual participant basis, ensuring the output is not influenced by the inter-site variation. Moreover, the statistical assessments of group differences were conducted on the normalized participant loadings, thereby disregarding any site-specific scaling differences in the analyses. Additionally, we addressed site differences by utilizing ComBat, which has been shown to demonstrate good performance in controlling for site effects [12]. The site differences in our study have been effectively considered while preserving biologically meaningful variations. Despite these efforts, it cannot be conclusively stated that the failure of replication is entirely unrelated to the site differences.

The ENIGMA sample used in the current study was selected to retain a similar demographic structure to that of the LEAP sample (i.e., with same age and IQ ranges). However, achieving full feasibility was not possible due to the absence of individuals in the control group of ENIGMA with an IQ below 70. Additionally, attempting to maintain a representative sample of autistic individuals, we avoided matching ENIGMA sample to LEAP. Consequently, there are notable differences in the descriptive distributions between the two samples (please see Additional file 1: Table S4). Moreover, in ENIGMA, limited information of the co-occurring psychiatric conditions (e.g., attention deficit hyperactivity disorder, anxiety) and medication use could impact the outcomes.

When site effects were effectively removed, the results between diagnostic groups significantly differed across sites in the current study. The discrepancy of results varies within sites may relate to the high clinical, biological, and etiologic heterogeneity within autistic individuals [17]. For our analysis, categorical groups were defined according to clinical diagnosis, which depended on the observations of the behaviors under a definition of autism in the developing diagnostic criteria (e.g., DSM-IV to DSM-5). The changing criteria unavoidably increased the heterogeneity within those with an autism diagnosis, at least at the level of behavioral manifestations, and likely also within research populations—especially those collected over longer periods. For instance, the DSM-5 criteria consolidated autism as a spectrum with excluding the language impairments and including altered sensory responses as a defined group of traits [2]. Additionally, biological phenotypes (endpoints), such as GM volume and behavioral phenotypes, can be traced back to diverse mechanisms (i.e., equifinality in the developmental psychopathology) [10, 27]. Moreover, the genetic contributions to autism are extremely complex. These include inherited and/or de novo genetic mutations, de novo or inherited copy number variations, common genetic variants, and associations with syndromal forms of autism [9, 25]. In addition, there are multiple other suggested factors, such as advanced maternal and paternal age and prenatal exposure to sex steroids, or medications like valproate [5]. The etiological and behavioral heterogeneity of autism increases the difficulty of identifying a general GM pattern for autism and of replicating them as average group effects may be hidden by individual variability varying between sites.

Our findings here reproduced the GM covariation spatial configurations that we found to be related to autism in LEAP. These covaried brain areas have been reported relating to autism widely in previous studies and have primarily been implicated in autism-related social and non-social behaviors [7, 13, 15, 16, 24]. The importance of the covariations of insula, IFG and OFC was emphasized in our multimodal study [21]. The gray matter covariations patterns of these regions shared variance with diffusion weighted imaging metrics and related to autism as well. In our previous study, we used a canonical correlation analysis to address within group variability and relate GM covariation patterns to various behavioral measures. Unfortunately, this was not an option with the ENIGMA data as only limited continuous measures of behavior were available.

On the one hand, big datasets, like ENIGMA, are exploited as the increased sample size improves the possibility of identifying effects with small effect size. However on the other hand, the retrospective pooling of data from multiple studies can introduce more heterogeneity than the increase in power can offset. While heterogeneity may seem like a hindrance to research it is not and it is important to involve a heterogenous ‘representative’ autistic sample with not only large sample size but multiple levels of data [17]. Utilizing multivariate/multimodal approaches in future studies and focusing on subtyping or continuous behavioral measures, rather than categorical group differences, may be more promising avenues for biomarker discovery and validation. Additionally, future studies dedicated to individual differences in autism probing heterogeneity could provide more insight for biomarker discovery, for example, normative modeling, which has been applied to the LEAP cohort [30]. The effect of group differences in cortical measure may vary across different subgroups.

Our findings should be interpreted at the background of several limitations. As we selected only participants with available IQ estimates, this probably induced some sample selection bias. Sex distribution, and IQ did not match across autistic and non-autistic groups, however we controlled for the effects of these two factors in the analyses. Moreover, the distributions of age, IQ and sex between LEAP and ENIGMA are statistically different from each other. Therefore, more validation analyses in the future will be done in the IQ and sex matched subsamples. Additionally, small samples and/or autism-only samples at multiple sites could potentially influence the outcome. Owing to limited access to the behavioral data of the autism group, we were unable to further our understanding of the dimensional behaviors of the sample.

In summary, the current study is unable to replicate the autism-related brain patterns from LEAP to ENIGMA cohorts. The diverse group effects across sites of ENIGMA demonstrate the challenges of generalizing the average findings of the GM covariation patterns to another large-scale cohort integrated retrospectively from multiple studies. Subsequent studies could utilize a sample with more comparable demographic information or prioritize subtyping to gain additional insights into the generalizability of these two GM covariation patterns.

### Supplementary Information

**Additional file 1.** Supplementary Tables and Figure.

## Data Availability

This study utilized 34 distinct datasets collected globally, each to diverse consent procedures and regulatory authorities. Access requests to these datasets will be evaluated in accordance with the corresponding consent agreements, guidelines, and regulations. You can submit requests through the ENIGMA consortium's ASD working group at http://enigma.ini.usc.edu/ongoing/enigma-asd-working-group/.

## Abbreviations

ADOS: Autism Diagnostic Observation Schedule–Generic
ENIGMA: Enhancing Neuroimaging Genetics Through Meta-Analysis
FWHM: Full-width half-max
GLM: Generalized linear model
GM: Gray matter
IC: Independent component
ICA: Independent component analysis
IFG: Inferior frontal gyrus
IQR: Image quality ratings
LEAP: Longitudinal European Autism Project
MRI: Magnetic resonance imaging
OFC: Orbitofrontal cortex
PHG: Parahippocampal gyrus
VBM: Voxel-based morphometry
