# Supplementary material for: Gray matter covariations in autism: out-of-sample replication using the ENIGMA autism cohort
Source: Mol Autism. 2024 Jan 17;15:3. doi: 10.1186/s13229-024-00583-8 (PMC10792893; doi:10.1186/s13229-024-00583-8)
Supplement: Supplementary file 1 — Additional file 1. Supplementary Tables and Figure. [file 13229_2024_583_MOESM1_ESM.pdf]

Supplementary Information

Gray Matter Covariations in Autism. Out of Sample Replication Using the ENIGMA-autism Cohort

Contents

1. Table S1. Acquisition Parameters in Each Site 2
2. Site Effect Removal Using ComBat and GLM 3
3. Table S2. Demographic Information of the Sample in Each Site 4
4. Comparison of the ICs between Groups in Each Site 6
5. Table S4. Comparison of Descriptive between ENIGMA and LEAP Sample 7

1. Table S1. Acquisition Parameters in Each Site

Site	Field Strength	Coverage	Slices	Voxel Size(mm)	TE
NIJMEGEN3	1.5T	256x256	176	1x1x1	2.3
BRC	3T	256x256	166	1x1x1	NA
PITT	3T	256x256	176	1.05x1.05x1.05	3.9
OHSU	3T	256x240	160	1x1x1	NA
ABIDE_CALTECH	3T	256x256	176	1x1x1	2.73
ABIDE_KKI	3T	256x256	200	1x1x1	3.7
ABIDE_LEUVEN_1	3T	256x256	182	1x1x1	4.6
ABIDE_MAX_MUN	3T	256x256	160	1x1x1	3.06
ABIDE_NYU	3T	256x256	128	1.3x1x1.3	3.25
ABIDE_OHSU	3T	256x256	160	1x1x1.1	3.58
ABIDE_OLIN	3T	256x256	176	1x1x1	2.74
ABIDE_PITT	3T	269x269	176	1x1x1	3.93
ABIDE_SDSU	3T	256x256	180	1x1x1	4.3
ABIDE_STANFORD	3T	256x256	132	0.9x0.9x1	1.8
ABIDE_UM_1	3T	256x256	128	1x1x1	1.8
ABIDE_UM_2	3T	256x256	128	1x1x1	1.8
ABIDE_USM	3T	256x240	192	1x1x1.2	2.91
ABIDE_YALE	3T	256x256	160	1x1x1	1.73
ABIDEII-BNI_1	3T	244x227	170	1.11x1.11x1.2	3.1
ABIDEII-ETH_1	3T	256x256	162	0.89x0.89x0.89	3.9
ABIDEII-GU_1	3T	256x256	176	1x1x1	3.5
ABIDEII-IP_1	1.5T	256x256	170	1x1x1	5.6
ABIDEII-IU_1	3T	256x256	180	0.7x0.7x0.7	2.3
ABIDEII-KKI_1	3T	256x200	200	1x1x1	3.7
ABIDEII-KUL_3	3T	256x256	182	1.2x1.2x1.2	4.6
ABIDEII-NYU_1	3T	256x256	128	1.3x1x1.3	3.25
ABIDEII-NYU_2	3T	256x256	128	1.3x1x1.3	3.25
ABIDEII-OHSU_1	3T	256x256	160	1x1x1.1	3.58
ABIDEII-OILH_2	3T	256x256	208	0.8x0.8x0.8	2.88
ABIDEII-SDSU_1	3T	256x192	176	1x1x1	3.172
ABIDEII-TCD_1	3T	256x256	190	0.9x0.9x0.9	3.9
ABIDEII-UCD_1	3T	256x256	192	1x1x1	3.16
ABIDEII-UCLA_1	3T	256x240	160	1x1x1.2	2.86
ABIDEII-USM_1	3T	256x256	220	1x1x1	NA

2. Site Effect Removal Using ComBat and GLM

We first attempted to remove the site effects by using ComBat, and by just adding site as a covariate in the GLM. The site removal effects were shown in Figure S1, which shows the density plots of participant loadings before and after controlling for site while using either a ComBat + GLM model or GLM alone. Both approaches effectively removed the site effect. The site effect was found significantly on the participant loadings of E-IC1 and E-IC2 while controlling for group, age, sex and IQ ($p<0.001$). ComBat (on E-IC1: $p=0.999$, on E-IC2: $p=0.868$) and GLM (on E-IC1: $p=0.999$, on E-IC2: $p=0.999$) were demonstrated effectively removing the site effect.

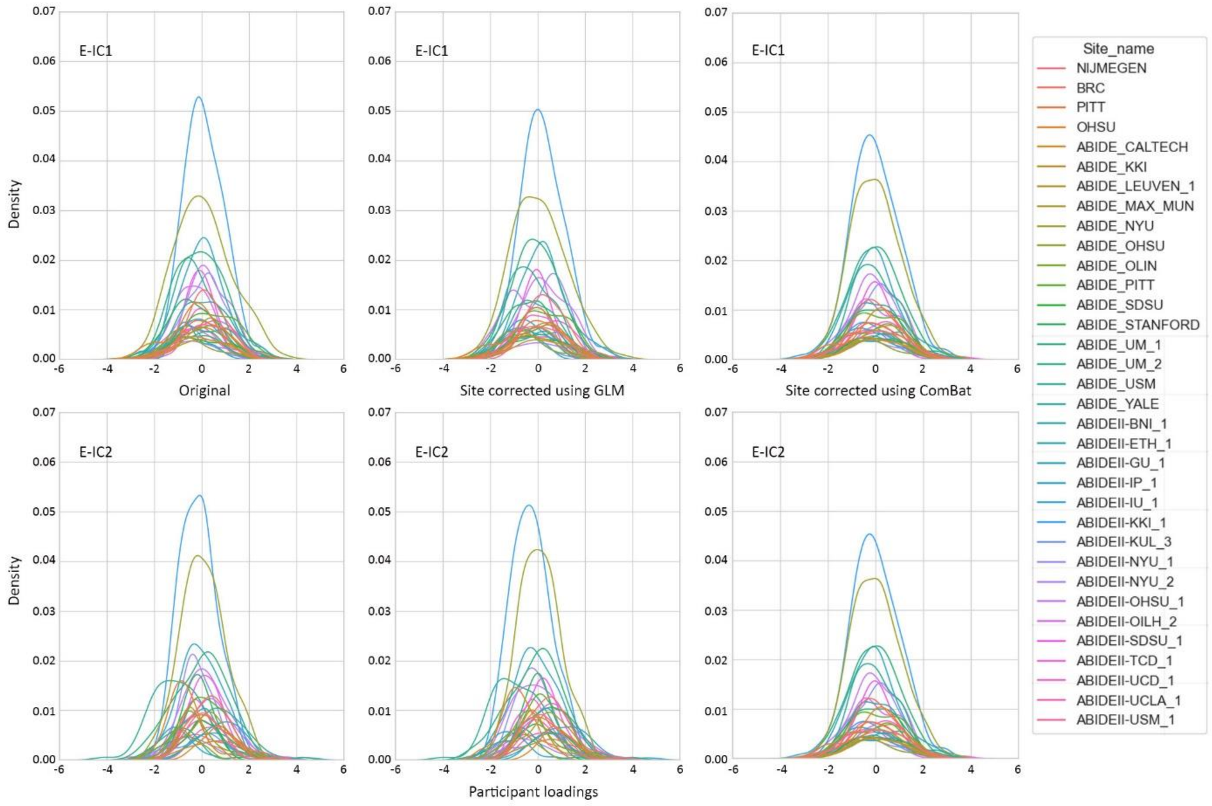

Figure S1. The kernel density estimation plot of participant loadings of each site before and after controlling for site separately using GLM and ComBat. E-IC, independent component of ENIGMA; L-IC, independent component of LEAP.

3. Table S2. Demographic Information of the Sample in Each Site

Site	Autism						Controls			
	n	Age (mean, SD)	Female	IQ (mean, SD)	ADOS, *n*	ADOS (mean, SD)	*n*	Age (mean, SD)	Female	IQ (mean, SD)
NIJMEGEN3	17	23.82 (3.99)	47.06%	117.76 (12.84)	-	-	15	22.27 (3.06)	40.00%	117.87 (12.49)
BRC	19	15.00 (1.94)	-	113.53 (15.68)	17	9.59 (5.03)	31	14.26 (2.63)	0.00%	110.26 (11.86)
PITT	17	15.18 (4.17)	11.76%	110.76 (9.32)	17	12.00 (3.74)	18	14.61 (3.96)	16.67%	109.94 (10.65)
OHSU	17	11.08 (2.21)	11.76%	104.47 (18.48)	10	6.60 (2.01)	28	10.93 (1.18)	28.57%	112.79 (14.76)
ABIDE_CALTECH	10	22.69 (3.48)	10.00%	108.20 (12.20)	10	11.60 (3.53)	11	22.95 (3.12)	36.36%	116.09 (10.34)
ABIDE_KKI	7	10.07 (1.13)	-	99.71 (16.37)	7	12.14 (3.02)	13	10.45 (1.46)	23.08%	113.46 (9.71)
ABIDE_LEUVEN_1	12	21.00 (3.13)	-	111.83 (12.32)	-	-	14	23.29 (3.02)	-	115.00 (13.32)
ABIDE_MAX_MUN	15	16.87 (8.37)	6.67%	105.00 (13.91)	6	9.50 (3.56)	22	21.55 (7.14)	4.55%	112.73 (8.72)
ABIDE_NYU	74	13.86 (5.92)	13.51%	107.66 (16.90)	74	11.15 (3.84)	100	16.08 (5.94)	26.00%	113.37 (13.32)
ABIDE_OHSU	7	10.85 (1.31)	-	95.73 (20.47)	7	8.57 (3.41)	10	9.70 (.98)	-	115.87 (12.03)
ABIDE_OLIN	18	16.33 (3.12)	11.11%	113.00 (17.87)	18	14.11 (3.91)	16	16.94 (3.68)	12.50%	114.94 (16.54)
ABIDE_PITT	24	17.04 (4.70)	16.67%	108.46 (13.31)	21	12.90 (3.05)	25	17.73 (5.41)	16.00%	109.72 (9.46)
ABIDE_SDSU	3	13.84 (1.64)	-	120.67 (7.57)	3	13.33 (5.13)	21	14.09 (1.85)	23.81%	107.67 (10.56)
ABIDE_STANFORD	23	17.20 (3.63)	-	108.87 (15.26)	23	10.65 (2.89)	29	16.17 (4.20)	-	111.14 (12.09)
ABIDE_UM_1	54	12.57 (2.98)	20.37%	100.75 (17.41)	8	14.25 (4.23)	54	12.88 (3.38)	35.19%	109.52 (10.14)
ABIDE_UM_2	11	14.34 (1.98)	-	106.77 (18.24)	2	18.00 (2.83)	12	16.33 (3.61)	8.33%	110.17 (9.97)
ABIDE_USM	49	19.04 (5.21)	-	99.41 (15.29)	49	12.98 (3.58)	34	19.43 (5.88)	-	114.21 (14.74)
ABIDE_YALE	27	12.61 (3.01)	29.63%	96.56 (19.21)	1	11.00 (-)	27	12.69 (2.80)	29.63%	105.00 (17.72)
ABIDEII-BNI_1	13	20.77 (2.20)	-	104.38 (15.33)	12	11.33 (3.96)	10	20.80 (2.57)	-	111.80 (10.32)
ABIDEII-ETH_1	11	20.56 (3.71)	-	107.55 (13.49)	10	9.00 (1.94)	24	23.88 (4.50)	-	116.50 (9.48)
ABIDEII-GU_1	44	11.01 (1.51)	15.91%	119.27 (14.81)	33	10.64 (4.31)	51	10.45 (1.74)	50.98%	121.63 (13.37)
ABIDEII-IP_1	15	15.36 (4.63)	33.33%	96.07 (22.93)	15	13.87 (4.56)	4	9.29 (.83)	75.00%	101.25 (25.70)
ABIDEII-IU_1	17	21.59 (3.30)	11.76%	115.76 (12.81)	17	8.82 (2.16)	17	21.94 (2.05)	29.41%	115.76 (9.88)
ABIDEII-KKI_1	53	10.34 (1.54)	26.42%	104.40 (15.19)	19	13.95 (3.31)	145	10.35 (1.19)	35.86%	114.47 (10.71)
ABIDEII-KUL_3	23	22.39 (3.31)	-	108.00 (15.91)	23	8.48 (3.86)	-	-	-	-
ABIDEII-NYU_1	37	10.22 (4.70)	8.11%	101.51 (19.80)	30	9.33 (3.09)	25	10.03 (3.37)	4.00%	117.52 (14.51)
ABIDEII-NYU_2	19	7.31 (0.78)	10.53%	104.37 (14.29)	1	14.00 (-)	-	-	-	-

Table S2. Demographic Information of the Sample in Each Site (Continued)

	Autism						Controls			
Site	*n*	Age (Mean, SD)	Female	IQ (Mean, SD)	ADOS, *n*	ADOS (Mean, SD)	*n*	Age (Mean, SD)	Female	IQ (Mean, SD)
ABIDEII-OHSU_1	26	12.50 (1.88)	19.23%	106.46 (17.65)	-	-	50	10.22 (1.63)	52.00%	117.72 (11.93)
ABIDEII-OILH_2	21	21.38 (3.22)	14.29%	113.38 (16.66)	21	9.43 (2.01)	34	24.03 (3.68)	41.18%	111.97 (12.18)
ABIDEII-SDSU_1	31	13.24 (3.07)	22.58%	99.48 (14.86)	-	-	24	13.13 (3.04)	8.33%	103.29 (11.86)
ABIDEII-TCD_1	17	14.40 (3.29)	-	108.94 (15.69)	17	8.71 (2.37)	21	15.65 (3.12)	-	116.29 (12.91)
ABIDEII-UCD_1	16	14.95 (1.96)	18.75%	104.00 (12.81)	1	8.00 (-)	14	14.80 (1.71)	28.57%	113.00 (11.23)
ABIDEII-UCLA_1	12	12.02 (2.23)	8.33%	104.33 (12.94)	8	12.88 (2.70)	13	9.99 (2.30)	38.46%	113.77 (14.21)
ABIDEII-USM_1	11	17.90 (5.16)	18.18%	97.45 (19.78)	6	15.50 (4.32)	12	22.38 (5.92)	25.00%	116.67 (15.56)

4. Comparison of the ICs between Groups in Each Site

Table S3. Comparison between Autism and Control Group in Each Site

Sites	E-IC1		E-IC2		Sites	E-IC1		E-IC2	
	t	*p* value	*t*	*p* value		*t*	*p* value	*t*	*p* value
NIJMEGEN	0.180	0.858	-0.564	0.577	ABIDE_YALE	-1.932	0.059	1.464	0.149
BRC	-0.819	0.417	-0.361	0.720	ABIDEII-BNI_1	-0.594	0.559	-0.224	0.825
PITT	-1.087	0.285	**2.302**	**0.028**	ABIDEII-ETH_1	0.512	0.612	0.055	0.957
OHSU	-0.362	0.719	0.824	0.415	ABIDEII-GU_1	0.229	0.820	-0.121	0.904
ABIDE_CALTECH	0.972	0.343	-0.221	0.828	ABIDEII-IP_1	0.165	0.871	1.104	0.285
ABIDE_KKI	0.349	0.731	0.279	0.783	ABIDEII-IU_1	0.123	0.903	0.798	0.431
ABIDE_LEUVEN_1	0.752	0.460	-1.252	0.223	ABIDEII-KKI_1	-0.441	0.660	0.503	0.615
ABIDE_MAX_MUN	1.871	0.070	1.457	0.154	ABIDEII-KUL_3	-	-	-	-
ABIDE_NYU	**4.499**	***p*<0.001**	-0.283	0.777	ABIDEII-NYU_1	-0.083	0.934	0.360	0.720
ABIDE_OHSU	0.115	0.910	1.405	0.181	ABIDEII-NYU_2	-	-	-	-
ABIDE_OLIN	**4.156**	***p*<0.001**	1.641	0.110	ABIDEII-OHSU_1	0.285	0.776	**2.964**	**0.004**
ABIDE_PITT	-1.718	0.092	-1.412	0.165	ABIDEII-OILH_2	1.882	0.065	0.568	0.573
ABIDE_SDSU	**-2.205**	**0.038**	-1.358	0.188	ABIDEII-SDSU_1	0.434	0.666	-0.373	0.710
ABIDE_STANFORD	**2.362**	**0.022**	0.653	0.517	ABIDEII-TCD_1	-0.881	0.384	-1.453	0.155
ABIDE_UM_1	**5.369**	***p*<0.001**	**4.880**	***p*<0.001**	ABIDEII-UCD_1	-0.713	0.482	0.872	0.391
ABIDE_UM_2	-0.859	0.400	-0.489	0.630	ABIDEII-UCLA_1	1.484	0.152	1.275	0.215
ABIDE_USM	0.042	0.966	-0.537	0.593	ABIDEII-USM_1	1.451	0.162	1.464	0.158

5. Table S4. Comparison of Descriptive between ENIGMA and LEAP Sample

	ENIGMA-Autism	LEAP-Autism	Statistics	ENIGMA-Control	LEAP-Control	Statistics
Diagnosis (N)	770	347		924	252	
Age (Mean, SD)	14.88 (5.47)	16.79 (5.56)	t=-5.374, p<0.001	14.79 (5.75)	16.92 (5.71)	t=-5.207, p<0.001
IQ (Mean, SD)	106.20 (16.91)	99.40 (18.94)	t=5.984, p<0.001	113.40 (12.74)	104.88 (18.26)	t=-6.954, p<0.001
Sex (Male, Female)	667,103	253, 94	χ^2=30.965, p<0.001	693, 231	163, 89	χ^2=10.641, p=0.001
